# Factors affecting quality of life in patients on haemodialysis: a cross-sectional study from Palestine

**DOI:** 10.1186/s12882-016-0257-z

**Published:** 2016-04-27

**Authors:** Sa’ed H. Zyoud, Dala N. Daraghmeh, Diana O. Mezyed, Razan L. Khdeir, Mayas N. Sawafta, Nora A. Ayaseh, Ghada H. Tabeeb, Waleed M. Sweileh, Rahmat Awang, Samah W. Al-Jabi

**Affiliations:** Poison Control and Drug Information Center (PCDIC), College of Medicine and Health Sciences, An-Najah National University, Nablus, 44839 Palestine; Department of Clinical and Community Pharmacy, College of Medicine and Health Sciences, An-Najah National University, Nablus, 44839 Palestine; WHO Collaborating Centre for Drug Information, National Poison Centre, Universiti Sains Malaysia (USM), Penang, 11800 Malaysia; PharmD program, College of Medicine and Health Sciences, An-Najah National University, Nablus, Palestine; Department of Pharmacology and Toxicology, College of Medicine and Health Sciences, An-Najah National University, Nablus, 44839 Palestine

**Keywords:** Haemodialysis, Risk factors, Health-related quality of life, Palestine

## Abstract

**Background:**

Haemodialysis (HD) is a life-sustaining treatment for patients with end-stage renal disease (ESRD). HD can bring about significant impairment in health-related quality of life (HRQOL) and outcomes. Therefore, we sought to describe the patterns of HRQOL and determine the independent factors associated with poor HRQOL in Palestinian patients on HD.

**Methods:**

A multicenter cross-sectional study was performed from June 2014 to January 2015 using the EuroQOL-5 Dimensions instrument (EQ-5D-5L) for the assessment of HRQOL. ESRD patients undergoing HD in all dialysis centres in the West Bank of Palestine were approached and recruited for this study. Multiple linear regression was carried out to identify factors that were significantly associated with HRQOL.

**Results:**

Two hundred and sixty-seven patients were participated in the current study giving response rate of 96 %. Overall, 139 (52.1 %) were male, and the mean ± standard deviation age was 53.3 ± 16.2 years. The reported HRQOL as measured by mean EQ-5D-5L index value and Euro QOL visual analogue scale (EQ-VAS) score was 0.37 ± 0.44 and 59.38 ± 45.39, respectively. There was a moderate positive correlation between the EQ-VAS and the EQ-5D-5L index value (*r* = 0.42, *p* < 0.001). The results of multiple linear regression showed a significant negative association between HRQOL with age, total number of chronic co-morbid diseases and the total number of chronic medications. However, a significant positive association was found between HRQOL with male gender, university education level and patients who live in village.

**Conclusion:**

Our results provided insight into a number of associations between patient variables and their HRQOL. Healthcare providers should be aware of low HRQOL among patients with no formal education, female gender, patient’s residents of refugee camps, multiple co-morbid diseases, multiple chronic medications, and elderly patients to improve their quality of life.

## Background

Chronic kidney disease (CKD) is a growing worldwide public health concern [1, 2]. It is characterised by an irreversible worsening of renal function that could lead to end-stage renal disease (ESRD), which necessitates treatment with renal replacement therapy (RRT) such as renal transplant or haemodialysis (HD) [3, 4]. HD is one of the most effective therapeutic techniques for patients with ESRD second to renal transplantation, but is expensive and burdensome therapy for patients with ESRD [1, 2, 5].

In the United States of America (USA), the number of newly reported ESRD cases in 2013 was 117,162 corresponding to an unadjusted incidence rate of 363 per million per year [6, 7]. In the USA, 88.2 % of all incident cases started with RRT with HD, 9.0 % initiated with peritoneal dialysis, and 2.6 % got a pre-emptive kidney transplant [6, 8]. In the West Bank of Palestine, the total number of patients with ESRD has increased notably over the last several years [9]. In 2014, the total reported number of ESRD patients undergoing HD in the West Bank was 1104 patients, representing an increase of 77.5 % compared to reported numbers in 2011 [10].

Previous reports have confirmed that ESRD patients undergoing HD have lower health-related quality of life (HRQOL) compared with compared with a normative sample [11–21]. Patients with ESRD undergoing HD are prone to several complications such as depression, inflammation, and malnutrition [21, 22]. Up to now, too little attention has been paid to document the HRQOL of patients with ESRD in the Middle East [12, 23, 24]. It has been shown that improving the quality of treatment in patients with ESRD can minimize the development and/or severity of complications and therefore improve patients’ HRQOL [25–27].

Health-related quality of life is a cultural concept as revealed by the difference in association between HRQOL and clinical outcomes such as compliance, or patient survival [28–30]. HRQOL is recognized as an essential health outcome for studies assessing the quality of healthcare, evaluating the impact of illness, and analyses of cost-effectiveness [31–33]. In addition, it has been shown that HEQOL is clinically important for improving dialysis outcome in patients on HD [34, 35].

Although several studies were carried out and published about HRQOL in different disease populations in Palestine such as diabetic or hypertensive patients [36, 37], no such studies were carried out among HD patients in Palestine. Therefore, we performed the present study to describe the patterns of HRQOL and to determine the independent factors associated with poor HRQOL in Palestinian patients with HD.

## Methods

### Study design

A multicenter cross-sectional study was carried out from June 2014 to January 2015.

### Study setting

Patients were recruited from all dialysis centres in West Bank, Palestine. We collated related information about the distribution of the population from the Palestinian Central Bureau of Statistics and Ministry of Health [38].

### Study population, sampling procedure and sample size calculation

The Healthcare sector in Palestine is primarily managed by the Government through the Palestinian Ministry of Health (PMOH). There are 10 functioning dialysis centres in West-Bank, Palestine. All the dialysis centres are on hospital campus and varies in size between one-machine to 32-machine centres. The PMOH runs nine out of the 10 available dialysis facilities [10]. At the time of the study, there were 740 dialysis patients served on 160 HD machines [38]. For the purpose of this study, sample size was detrmined using a Raosoft sample size calculator, which is a web-based calculator [39]. A sample size of 254 patients was considered to achieve a 5 % margin of error and a 95 % confidence level assuming that 50 % of patients answered each question correctly. The sample size was increased by 5–10 % to account for the non-response rate. Two hundred and seventy seven patients were selected using a convenience quota sampling method proportional to the number of patients in each dialysis centre. The inclusion criteria were as follows: (1) patients 18 years of age or older; (2) confirmed diagnosis of ESRD by medical file; and (3) on regular HD therapy for a minimum of 3 months prior to the interview. Patients were excluded if they lacked the mental or physical capacity to communicate with interviewer.

### Data collection instrument

Data were collected using a questionnaire containing two sections, a socio-demographic and clinical history section and a validated Arabic version of HRQOL section [40]. Details regarding age, gender, body mass index (BMI) quartiles calculated from height and weight, educational level, household monthly income were obtained, residency, living status, smoking status [“light smoker”: (1 to 9 cigarettes/day), “moderate smoker”: (10 to 19 cigarettes/day), and “heavy smoker”: (≥20 cigarettes/day) [41]], marital status, occupation, dialysis vintage (length of time on dialysis treatment), average duration of dialysis session, total number of medications for chronic use, and the total number of chronic diseases. We categorised BMI as obese (BMI ≥ 30 kg/m^2^), overweight (BMI = 25 to <30 kg/m^2^), normal (BMI = 18.5 to <25 kg/m^2^), or underweight (BMI < 18.5 kg/m^2^) [42]. To determine the health status, the 5-level EuroQoL Group’s 5-dimension (EQ-5D-5L) questionnaire was used. The EQ-5D instrument was developed by the Euro QOL Group. The EQ-5D includes a 5-item descriptive system to calculate the EQ-5D index score and the EQ visual analogue scale (EQ-VAS) that allows the patients to judge their current health status during intradialysis from 0 to 100. The measuring principle of the instrument was described in detail in the previous works by the investigators [36, 37, 43]. The Arabic version of EQ-5D [40] was provided by using Euro QOL guidelines [44]. The study was registered with Euro QOL and permission was given for its use (ID: 8537, approval date: April 7, 2014).

### Data collection procedure

Two hundred and seventy seven patients were recruited and interviewed face to face. We tried to make each interviewee feel as comfortable as possible. Face-to-face interview was used to provide researchers with an opportunity to collect as complete data as possible and to overcome non-response by those who cannot read. Interviews were performed by trained clinical pharmacy students. Data collection method was pre-tested to check for the clarity of questions in a pilot study of 16 patients was tested on eight medical files that were not included in the final anslysis. All the comments noted in the pilot study were taken into consideration and a modified questionnaire was reviewed by experts in the field of quality of live (QOL) to ensure content validity of data collection form in relations to factors that might be associated with QOL in ESRD population. The internal consistency of the EQ-5D instrument was found to be 0.84 which showed high reliability of the EQ-5D instrument.

### Ethical approval

The study protocol was approved by the Ethics Committee of An-Najah National University, and the local health authorities that had jurisdiction over the local study population. The interview content was described to respondents, and an informed verbal consent was obtained before the start of the interview.

### Statistical analysis

Data were analysed using SPSS (SPSS Inc., Chicago, IL, USA) programme version 15. Results were reported as mean ± SDs or as frequencies and percentages or as a median with a range of values (lower-upper quartiles) wherever appropriate. Data that were not normally distributed were analysed using the Mann-Whitney *U* test or Kruskal-Wallis test according to the number of groups to compare. The Kolmogorov-Smirnov test was used to assess normality of distribution of data. The Pearson correlation coefficient was used to assess the correlation between the reported EQ-VAS scores and EQ-5D-5L index values. Multiple linear regression was carried out to identify factors that were significantly associated with HRQOL. Multiple linear regression was carried out to identify factors that were significantly associated with HRQOL (dependent factor). The independent factors were socio-demographic, and HD related clinical factors. A dummy coding of 0 and 1 was used to enter the nominal independent variables such as gender, BMI, educational level, residency, and occupation into the regression model. Variables with a *p* < 0.05 in univariate analysis were entered in the regression model. The significance level was predetermined at p level < of 0.05 for all tests. Variance inflation factors (VIF) and tolerance index were conducted to assess collinearity between independent variables. The internal consistency reliability of the study scale was assessed using Cronbach’s alpha values. EQ-5D was scored to calculate the index value using the value sets (weights) from the existing United Kingdom general population scoring algorithm (i.e. EQ-5D-5L Crosswalk Index Value Calculator [45]). We used the UK value set for three reasons; first, to present the health status as a continuous variable; second, due to absence of a locally or regionally appropriate set of values; and lastly to make the comparison more reasonable because most published studies at local or regional level used the UK value set as suggested by EuroQol Group the EQ-5D [12, 37, 46].

## Results

### Socio-demographic and clinical characteristics

Two hundred and sixty-seven patients were participated in the current study giving response rate of 96 %. Overall, 139 (52.1 %) were male, and the mean (standard deviation) age was 53.3 (16.2) years. 177 patients (66.3 %) were on dialysis for less than four years. There were 204 patients (76.4 %) who were dialysed three times weekly among which 198 patients (74.2 %) stayed on dialysis three hours. The mean duration of disease was 3.4 ± 3.7 year. The majority of patients 197 (73.8 %) took their medication by themselves, 116 (43.4 %) had three or more chronic co-morbid diseases, and 222 (83.1 %) were on four or more chronic medications. The mean number of chronic co-morbid diseases was 2.4 ± 1.6 and the mean number of chronic medications was 6.5 ± 2.8. Overall, 52.7 % of study participants were either overweight or obese (28.1 % were overweight and 24.6 % were obese). Being obese was significantly more prevalent in females (14.8 %) as compared to males (9.8 %); (p value = 0.042). The socio-demographic and clinical characteristics of the study participants are displayed in Table 1.

**Table 1:** Socio-demographic and clinical characteristics of the study sample

Variable	Frequency (%) N (267)
Age category	
< 30	34 (12.7)
30–60	136 (50.9)
> 60	97 (36.3)
Gender	
Male	139 (52.1)
Female	128 (47.9)
BMI^a^	
Underweight	24 (9)
Normal	97 (36.3)
Overweight	72 (27)
Obese	63 (23.6)
Education	
No formal education	40 (15.0)
Primary	71 (26.6)
Secondary	102 (38.2)
Graduated	54 (20.2)
Household income (month)	
High (more than 1000 JD^b^)	10 (3.7)
Moderate (400–1000 JD)	90 (33.7)
Low (less than 400 JD)	167 (62.5)
Living	
Palestinian refugee camps	22 (8.2)
Village	161 (60.3)
City	84 (31.5)
Living status	
Alone	18 (6.7)
With family	249 (93.3)
Marital status	
Single, divorced, widowed	79 (29.6)
Married	188 (70.4)
Occupation	
Employed	35 (13.1)
Unemployed	232 (86.9)
Current smoking status	
Non smoker	227 (85)
Light smoker	19 (7.1)
Moderate smoker	17 (6.4)
Heavy smoker	4 (1.5)
Dialysis vintage (Years)	
< 4	177 (66.3)
≥ 4	90 (33.7)
Dialysis per week	
≤ 2	26 (9.7)
3	204 (76.4)
≥ 4	37 (13.9)
Dialysis session duration (hours)	
< 4	198 (74.2)
≥ 4	69 (25.8)
Transplantation history	
Yes	26 (9.7)
No	241 (90.3)
Total chronic co-morbid diseases	
None	26 (9.7)
1	62 (23.2)
2	63 (23.6)
≥ 3	116 (43.4)
Chronic medications (per day)	
< 4	45 (16.9)
≥ 4	222 (83.1)

*Abbreviations*: *BMI* body mass index, *JD* Jordanian Dinar

^a^Data were missing from 11 patients

^b^1 Jordanian Dinar (JD) equals 1.41 US Dollar

### EQ-5D health status

**Fig. 1:**
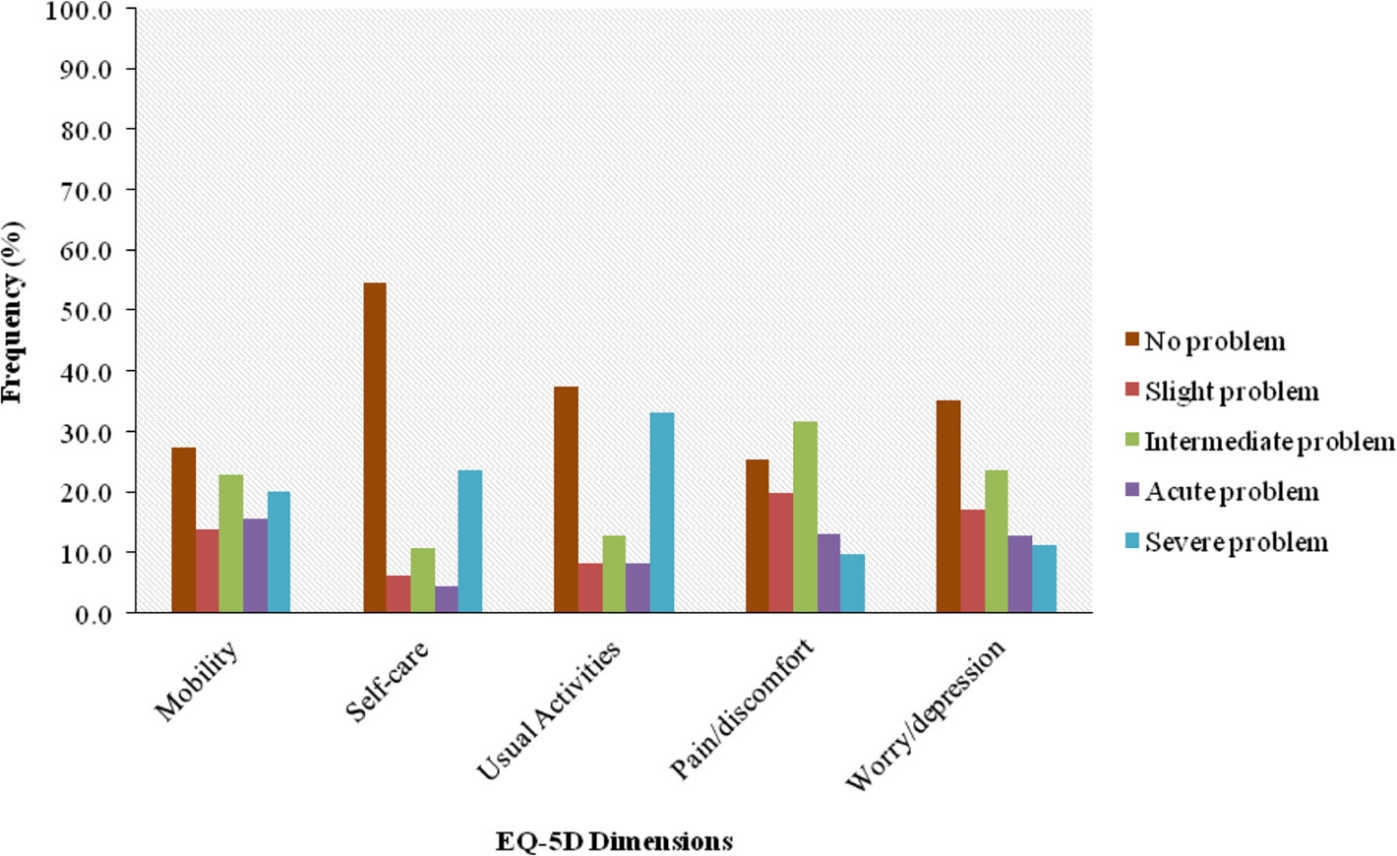
Distribution of health-related quality of life measures in different European Quality of Life scale 5 (EQ-5D) dimensions

The reported HRQOL as measured by mean EQ-5D-5L index value and EQ-VAS score was 0.37 ± 0.44 and 59.38 ± 45.39, respectively. There was a moderate positive correlation between the EQ-VAS and the EQ-5D-5L index value (*r* = 0.42, *p* < 0.001). The distribution of reported no problems across dimensions of QOL was as follows: mobility 73 (27.3 %), usual activities 100 (37.5 %), self-care 146 (54.7 %), pain/discomfort 68 (25.5 %) and anxiety/depression 94 (35.2 %); (Fig. 1). A total of 178 states of health were reported by the participants. We found that 17 (6.4 %) participants reported no problems for any dimension, and 9 (3.4 %) patients reported very severe difficulty for all five dimensions.

### EQ-5D-5L index values

The median EQ-5D-5L index value was 0.41 (interquartile range: 0.06-0.77). Tables 2 showed that there were significant differences between participant groups according to age, BMI, education level, residency and total co-morbid disease, as well as gender, occupation, and total number of chronic medication (p-value < 0.05). No significant differences were found between participants according to income, living status, marital status, dialysis vintage, dialysis session duration, smoking status, and transplantation history.

**Table 2:** EQ-5D total score by socio-demographic and clinical variables (*n* = 267)

Variable	Frequency (%) *N* = 267	EQ-5D index score		*P*-value
		Mean ± SD	Median [interquartile range]	
Age category				
< 30	34 (12.7)	0.69 ± 0.28	0.79 [0.47–0.88]	0.001^a^
30–60	136 (50.9)	0.44 ± 0.43	0.55 [0.18–0.77]	
> 60	97 (36.3)	0.17 ± 0.40	0.15 [0.13–0.56]	
Gender				
Male	139 (52.1)	0.46 ± 0.41	0.85 [0.22–0.80]	0.001^b^
Female	128 (47.9)	0.28 ± 0.45	0.21 [0.04–0.73]	
BMI^c^				
Underweight	24 (9.0)	0.57 ± 0.42	0.69 [0.38–0.86]	0.047^a^
Normal	97 (36.3)	0.41 ± 0.42	0.46 [0.10–0.78]	
Overweight	72 (27)	0.36 ± 0.45	0.43 [0.01–0.75]	
Obese	63 (23.6)	0.29 ± 0.43	0.26 [0.03–0.73]	
Education				
No formal education	40 (15.0)	0.04 ± 0.41	0.06 [−0.21–0.22]	<0.001^a^
Primary	71 (26.6)	0.36 ± 0.42	0.32 [0.08–0.74]	
Secondary	102 (38.2)	0.45 ± 0.42	0.55 [0.16–0.79]	
Graduated	54 (20.2)	0.50 ± 0.40	0.67 [0.23–0.84]	
Household income (month)				
High (more than 1000 JD^d^)	10 (3.7)	0.34 ± 0.48	0.28 [0.12–0.84]	0.109^a^
Moderate (400–1000 JD)	90 (33.7)	0.45 ± 0.43	0.64 [0.15–0.84]	
Low (less than 400 JD)	167 (62.5)	0.33 ± 0.44	0.36 [0.03–0.72]	
Residency				0.004^a^
Palestinian refugee camps	22 (8.2)	0.20 ± 0.39	0.15 [0.02–0.52]	
Village	161 (60.3)	0.44 ± 0.44	0.58 [0.15–0.78]	
City	84 (31.5)	0.29 ± 0.44	0.26 [0.02–0.73]	
Living status				
Alone	18 (6.7)	0.31 ± 0.37	0.28 [0.10–0.67]	0.347^b^
With family	249 (93.3)	0.38 ± 0.44	0.43 [0.07–0.77]	
Marital status				
Single, divorced, widowed	79 (29.6)	0.42 ± 0.44	0.48 [0.12–0.79]	0.171^b^
Married	188 (70.4)	0.35 ± 0.44	0.36 [0.07–0.75]	
Occupation				
Employed	35 (13.1)	0.51 ± 0.38	0.66 [0.21–0.88]	0.041^b^
Unemployed	232 (86.9)	0.35 ± 0.44	0.38 [0.06–0.75]	
Smoking				
Non-smoker	227 (85)	0.36 ± 0.44	0.36 [0.04–0.76]	0.156^a^
Light smoker	19 (7.1)	0.49 ± 0.48	0.64 [0.14–0.88]	
Moderate smoker	17 (6.4)	0.53 ± 0.38	0.66 [0.28–0.78]	
Heavy smoker	4 (1.5)	0.15 ± 0.46	0.10 [0.27–0.61]	
Dialysis session duration (hours)				
< 4 h	198 (74.2)	0.37 ± 0.45	0.41 [0.05–0.77]	0.859^b^
≥ 4 h	69 (25.8)	0.39 ± 0.42	0.41 [0.14–0.73]	
Dialysis vintage (years)				
< 4	177 (66.3)	0.37 ± 0.44	0.39 [0.08–0.76]	0.806^b^
≥ 4	90 (33.7)	0.38 ± 0.44	0.47 [0.04–0.77]	
Transplantation				
Yes	26 (9.7)	0.52 ± 0.42	0.66 [0.33–0.84]	0.055^b^
No	241 (90.3)	0.36 ± 0.44	0.35 [0.05–0.75]	
Total chronic co-morbid diseases				
None	26 (9.7)	0.77 ± 0.29	0.81 [0.69–1.00]	<0.001^a^
1	62 (23.2)	0.56 ± 0.39	0.69 [0.30–0.84]	
2	63 (23.6)	0.41 ± 0.34	0.38 [0.18–0.73]	
≥ 3	116 (43.4)	0.16 ± 0.43	0.15 [0.17–0.52]	
Chronic medications				
< 4 drugs/day	45 (16.9)	0.53 ± 0.37	0.67 [0.22–0.80]	0.010^b^
≥ 4 drugs/day	222 (83.1)	0.34 ± 0.44	0.33 [0.02–0.74]	

*Abbreviations*: *EQ-5D* European Quality of Life scale 5 dimensions, *JD* Jordanian Dinar, *BMI* body mass index

^a^Statistical significance of differences calculated using the Kruskal-Wallis test

^b^Statistical significance of differences calculated using the Mann-Whitney *U* test

^c^Data were missing from 11 patients

^d^1 Jordanian Dinar (JD) equals 1.41 US Dollar

### EQ-VAS score

The median EQ-VAS score was 50 (interquartile range: 50–70). As seen in Tables 3, there were significant differences between participant groups according to age and total co-morbid diseases, as well as gender, and total number of chronic medications (p-value < 0.05). Patients older than 60 years had a lower EQ-VAS score than those younger than 60. In addition, male gender was associated with higher EQ-VAS value compared to female. The study found that EQ-VAS score decreased as the total number of chronic medication increased and as illustrated in Table 3 increased co-morbid diseases had the lowest EQ-VAS score.

**Table 3:** EQ-VAS by socio-demographic and clinical characteristics

Variable	EQ-VAS		*P*-value
	Mean ± SD	Median [interquartile range]	
Age category			
< 30	59.95 ± 21.53	60 [50–80]	0.010^a^
30–60	60.28 ± 21.66	60 [50–75]	
> 60	51.46 ± 23.20	50 [30–70]	
Gender			
Male	61.26 ± 19.94	60 [50–80]	0.002^b^
Female	52.41 ± 24.28	50 [40–70]	
BMI^c^			
Underweight	66.13 ± 16.44	65 [50–80]	0.198^a^
Normal	55.98 ± 21.92	60 [45–70]	
Overweight	55.31 ± 24.68	50 [40–70]	
Obese	58.25 ± 20.81	50 [45–70]	
Smoking			
Non smoker	55.96 ± 22.63	50 [45–70]	0.294^a^
Light smoker	64.47 ± 21.07	70 [50–80]	
Moderate smoker	63.24 ± 22.29	60 [50–80]	
Heavy smoker	55.00 ± 20.82	55 [35–75]	
Education level			
No formal education	50.63 ± 23.62	50 [40–70]	0.111^a^
Primary	54.72 ± 24.20	50 [40–70]	
Secondary	59.79 ± 20.87	60 [50–70]	
Graduated	59.54 ± 21.77	63 [50–80]	
Household income (month)			
High (more than 1000 JD^d^)	58.50 ± 20.00	55 [45–80]	0.968^a^
Moderate (400–1000 JD)	56.63 ± 22.05	58 [49–70]	
Low (less than 400 JD)	57.14 ± 23.03	50 [50–72]	
Residency			
Palestinian refugee camps	62.5 ± 21.92	55 [50–80]	0.145^a^
Village	58.25 ± 21.99	60 [50–74]	
City	53.21 ± 23.38	50 [40–70]	
Living statues			
Alone	57.50 ± 21.30	60 [50–70]	0.761^b^
With family	56.98 ± 22.65	50 [48–72]	
Marital status			
Single, divorced, widowed	55.78 ± 21.84	50 [50–70]	0.608^b^
Married	57.54 ± 22.85	60 [50–71]	
Occupation			
Employed	58.57 ± 21.54	60 [45–75]	0.509^b^
Unemployed	56.78 ± 22.71	50 [50–70]	
Dialysis session duration (hours)			
< 4 h	59.16 ± 51.11	50 [40–70]	0.163^b^
≥ 4 h	60.01 ± 22.13	60 [50–74]	
Dialysis vintage (years)			
< 4	55.86 ± 22.45	50 [50–70]	0.275^b^
≥ 4	66.30 ± 71.33	60 [49–80]	
Transplantation			
Yes	62.58 ± 19.54	68 [50–76]	0.212^b^
No	59.04 ± 47.35	50 [45–70]	
Total chronic co-morbid diseases			
None	63.15 ± 21.53	60 [50–80]	0.001^a^
1	62.82 ± 21.75	63 [50–80]	
2	59.68 ± 21.04	65 [50–80]	
≥ 3	56.53 ± 64.48	50 [40–70]	
Chronic medications			
< 4 drugs/day	63.56 ± 19.96	60 [50–80]	0.042^b^
≥ 4 drugs/day	58.54 ± 48.95	50 [40–70]	

*Abbreviation*: *EQ-VAS* European Quality visual analogue scale, *JD* Jordanian Dinar, *BMI* body mass index

^a^Statistical significance of differences calculated using the Kruskal-Wallis test

^b^Statistical significance of differences calculated using the Mann-Whitney *U* test

^c^Data were missing from 11 patients

^d^1 Jordanian Dinar (JD) equals 1.41 US Dollar

After adjustment for covariates, regression coefficients indicated significant associations between some of the independent variables and EQ-5D index score in comparison to a reference category for categorical variables or with one unit increase of a continuous variable. This model explained about 37 % of the variance in EQ-5D scores. As shown in Table 4, age, total number of chronic comorbid diseases and the total number of chronic medications were negatively associated with EQ-5D scores, whereas male gender, university education level and patients who live in village were positively associated with the EQ-5D scores. The range of VIF was from 1.015 to 1.465 which indicated absence of multicollinearity between independent variables.

**Table 4:** Multiple linear regression analysis of association between factors and EQ-5D score

Variables	Unstandardised coefficients (B)	Standardised coefficients (Beta)	P value	95 % CI for B
Age				
Continuous (1-year units)	−0.007	−0.272	<0.001	−0.010– −0.004
Gender				
Male	0.154	0.177	0.001	0.064–0.244
Female	Reference			
BMI				
Underweight or normal	−0.034	−0.039	0.464	−0.124–0.057
Overweight or obese	Reference			
Education level				
University	0.119	0.109	0.042	0.005–0.233
No formal, primary, secondary	Reference			
Residency				
Village	0.121	0.136	0.007	0.033–0.210
Palestinian refugee camps or city	Reference			
Occupation				
Employed	0.009	0.007	0.892	−0.124–0.142
Unemployed	Reference			
Number of co-morbid diseases				
Continuous	−0.089	−0.324	<0.001	−0.121– −0.056
Number of chronic medications				
Continuous	−0.018	−0.113	0.039	−0.034– −0.001

*Abbreviations*: *EQ-5D* European quality of life scale, *SE* standard error, *BMI* body mass index, *CI* confidence interval

## Discussion

This study provided a comprehensive analysis of HRQOL among ESRD patients undergoing HD in the West Bank of Palestine. HRQOL was assessed using the EQ-5D Overall, this study indicated that the main sociodemographic factors associated with HD-related QOL were old age, female gender, obesity, residency in a refugee camp, unemployment, low income, and having no formal education. Review of literature indicated that the EQ-5D has been used to measure HRQOL among ESRD patients undergoing HD in different countries [11–19]. The construct validity, reliability, and responsiveness of the EQ-5D have been recognized widely in both specific and general disease populations [47, 48]. Furthermore, Wasserfallen et al. [49] showed that using a generic QOL instrument EQ-5D was well-accepted, and easy to use for assessing HRQOL among ESRD patients undergoing HD due to the shorter completion time compared with other generic instruments.

In the current study, we found that mean EQ-5D score among ESRD patients undergoing HD was 0.37 ± 0.44 while findings from studies that used the same instruments in Korean, Japanese, Taiwan, and Singaporean patients were 0.704 ± 0.199 [17], 0.75 ± 0.17 [18], 0.65 ± 0.23 [13], and 0.60 ± 0.21 [20], respectively. Several socioeconomic and healthcare system related factors could affect HRQOL among ESRD patients undergoing HD. Some of these variations in EQ-5D score could be explained by differences in the main sociodemographic and clinical characteristics of recruited participants such as; age, duration of HD and presence of comorbid diseases. Furthermore, many patients, particularly in developing countries, frequently do not seek medical advice until other debilitating symptoms or complications appeared, thus, delay in diagnosis and therapy can directly increase the number of complications and therefore leading to reduction in patient’s HRQOL [50].

In our study, there was a modest positive correlation between the EQ-5D index values and reported EQ-VAS scores. Several studies suggested that individual experiences that are assessed by different rating scales such as the EQ-5D-5L and EQ-VAS may result in slightly different outcomes [12, 51, 52]. In addition, Saffari et al. [12] declared that when contributors were asked to select their health status using five dimensions, accuracy in outcomes is probable than when using only one overall dimension of health status.

Our results demonstrated that increased age was associated with lower HRQOL. Similar previous studies have reported the same findings, for instance, Kang et al.’s [17] study using EQ-5D found age was a significant factor determining HRQOL of Korean HD patients. Younger patients (<30 years) in the current study reported significantly better HRQOL, possibly because of the short duration of disease, and minor complications [37]. According to another study, older age was the most important predictor of lower QOL and health status [12]. Our study found that female gender was significantly associated with lower mean EQ-5D scores than male gender. One possible explanation is that poor social life and physical inactivity of females in developing countries might contribute to lower QOL scores, thus, females tend to have poor QOL [50]. This observation is in agreement with Merom et al [53] findings which identified Palestinian women as being at the highest risk of physical inactivity. Furthermore, males were less likely to become anxious or depressed compared to females [12, 54], thus, patients presented with more symptoms of depression and anxiety indicated lower levels of QOL [55–58]. The other possible explanation for this result may be that females were more obese in our study, which by itself worsens HRQOL, as reported by Bossola et al. [59] and Feroze et al. [54, 60]. According to our study, obese patients were significantly associated with lower EQ-5D scores. In the USA, a study conducted by Dwyer et al. [61] also mentioned obesity as one of the factors associated with impaired HRQOL and recommended the importance of keeping weight at healthier levels for improvement of QOL.

This study found significant associations between high education level and high HRQOL. This could be due to the fact that educated patients may have a better understanding of the illness, its effects, and will themselves benefit from the best management they can give [50], or they have more information about the treatments, greater self reported adherence, and a better relationship with their healthcare team [58]. Education was also confirmed in several studies as an important discriminator of HRQOL in HD patients [13, 62–64]. Our data showed that being unemployed was significantly related to lower EQ-5D scores. These results were in agreement with the findings documented by Sakthong and Kasemsup [13]. Unemployment was also confirmed in several studies as an important factor associated with impaired HRQOL in HD patients [23, 32, 58].

According to our study, residency in a refugee camp was also associated with low QOL with those living in a village had the highest EQ-5D index. These results further support the idea of closer communications and stronger family ties among people in rural areas as found by Saffari et al. [12] among Iranian population. A Lebanese study demonstrated that rural residents had higher vitality scores than urban residents [65]. However, it is hard to compare our results with other studies since the health care systems are different in different countries. Most of Palestinians in refugees in the camps receive their care from the UNRWA, and according to Eljedi et al. [66], patients treated at the UNRWA clinics may have a poorer quality of health care than patients getting care from other providers. Residency in refugee camps, as an important factor associated with impaired HRQOL, was also confirmed in several previous studies among different populations from Palestine such as diabetic or hypertensive patients [36, 37, 43].

As for clinical factors, presence of co-morbid diseases and increasing in the total number of medications have been recognised as variables that were negatively associated with HRQOL. Presence of co-morbidity was negatively associated with HRQOL. Similar associations were observed in previous studies [12, 20, 24, 63]. Chronic illnesses, mainly DM, were strongly associated with impaired HRQOL in ESRD patients on dialysis [67]. The number of medications was significantly associated with lower EQ-5D scores. These results were in agreement with the findings reported by Chiu and colleagues whereby people who took a large number of medications rated their health as poorer than those who did not [68]. A negative impact of medication on HRQOL might be mediated by the effect of medication-taking on patients’ behaviour due to high expense or side effects.

### Strengths and limitations

This study had many strengths such as including a generalised sample of all HD centres in the West Bank, as well as conducting face-to-face interviews to obtain more complete data and high reliability of data collection. Furthermore, our study is the first study assessing HRQOL among ESRD patients in the West Bank and to the best of our knowledge is the first one in Palestine that used the EQ-5D scale as a measure. However, there were a number of limitations that need to be noted. First, the cross-sectional nature of this study makes it difficult to interpret any cause–effect relationship. Second, we used a convenience sampling technique that could decrease the generalisability of the results to other HD patients. Third, data were collected via a face-to-face interview which might have introduced interviewer’s bias in the results. Lastly, additional clinical variables such as albumin, calcium, and creatinine would help to get a more complete view of possible dialysis outcome factors related to HRQOL of HD patients.

## Conclusions

Our results provide insight into a number of associations between patient variables such as demographics, clinical factors, and their HRQOL. Our research study reveals a number of important results that can be taken into consideration when dealing with HD patients. Elderly patients, female gender, obese patients, patients with no formal education, and living in Palestinian refugee camps were all associated with poor HRQOL. In addition, this study showed that lower HRQOL was associated with higher numbers of chronic diseases as well as higher numbers of medications. These results are expected to be of interest to educators, pharmacists, and clinicians working with ESRD patients. Healthcare providers should be aware of low HRQOL among patients with no formal education, female gender, patient’s residents of refugee camps, multiple co-morbid diseases, multiple chronic medications, and elderly patients to improve their QOL.

### Ethics approval and consent to participate

The study protocol was approved by the Ethics Committee of An-Najah National University. The interview content was described to respondents, and an informed verbal consent was obtained before the start of the interview.

### Consent for publication

Not applicable.

### Availability of supporting data

All data supporting the study is presented in the manuscript or available upon request from the corresponding author of this manuscript, Zyoud S. H.

## Abbreviations

CKD: Chronic kidney disease
EQ-5D-5L: 5-level EuroQoL Group’s 5-dimension
EQ-VAS: EuroQoL visual analogue scale
ESRD: end-stage renal disease
HD: haemodialysis
HRQOL: health-related quality of life, PMOH, Palestinian Ministry of Health, BMI, body mass index
QOL: quality of live
RRT: renal replacement therapy
SD: standard deviation
UNRWA: United Nations Relief and Works Agency for Palestine Refugees in the Near East
VIF: variance inflation factor
